# Sustainable gypsum binders reinforced with industrial and bio-waste additives

**DOI:** 10.1371/journal.pone.0357027

**Published:** 2026-08-27

**Authors:** Ahmed H. Mohammed, Ibrahim A. Atiyah, Dalia A. Rasool, Ahmed S. Abbas, Samer S. Abdulhussein, Ali A. Salman, Ali M. Flayyih, Kadhim K. Resan, Mohammed Ali Abdulrehman

**Affiliations:** 1 Department of Materials Engineering, College of Engineering, Mustansiriyah University, Baghdad, Iraq; 2 Department of Civil Engineering, College of Engineering, Al-mustafa University, Baghdad, Iraq; 3 Department of Civil Engineering, College of Engineering, Mustansiriyah University, Baghdad, Iraq; Coimbatore Institute of Technology, INDIA

## Abstract

This study aims to improve the mechanical strength, water resistance, and setting behavior of gypsum binders by adding industrial and agricultural waste. This study investigated the effects of ground orange peel powder, sodium silicate solution, and reactive silica on the functionality of gypsum composites intended for resource-efficient construction applications. Reactive silica (5–10%), GOPP (0–2%), and 20% sodium silicate solutions were added in different amounts to create nine mixtures. The compressive and flexural strengths, water absorption, and setting times were assessed. Scanning electron microscopy was used to monitor the microstructural development and comprehend the interactions between the additives and the gypsum matrix. The results showed that the ideal mixture (G7) consisting of 10% reactive silica, 20% sodium silicate solution, and 1% GOPP had a compressive strength of 16.9 MPa, flexural strength of 2.35 MPa, and low water absorption value of 5.41%. The GOPP successfully postponed the setting, enabling extended matrix densification and C-S-H formation. SEM analysis verified that the modified samples yielded better internal cohesion and lower porosity. This study presents a novel approach that combines organic and inorganic waste materials to enhance gypsum performance. GOPP offer waste-derived set-retarding qualities, in contrast to conventional chemical additives. The suggested process shows how gypsum properties can be improved using waste materials produced by various industries and agriculture.

## 1. Introduction

During construction, the evolution of binding materials reflects the persistent desire for high-performance options aligned with sustainability. Gypsum binders, which are traditionally favored for their workability and physical characteristics, have been widely used in construction and restoration. However, enhancing their mechanical properties remains a challenge, particularly owing to their demanding structural roles. Recent studies have explored supplementary materials to bolster the durability and strength of gypsum binders, addressing their inherent brittleness [1–3]. For instance, in one study by Kamarou et al. [4], the use of waste-based mineral admixtures was found to have a positive effect on the waterproofing and mechanical properties of gypsum binder formulations. This study focuses on the potential of reactive silica, sodium silicate solution, and citrus peel powder (a natural retarder) to improve gypsum binder mechanics, addressing the limitations that often hinder load-bearing applications [5–7]. In particular, this study sought to understand how these additives interact with the gypsum matrix. The key goals include evaluating the compressive and flexural strengths, setting times, and long-term durability of the modified binders [8–10]. Furthermore, the effectiveness of citrus peel powder as a retarder and in enhancing the microstructure of the binder was investigated. This promotes sustainable construction by upcycling agricultural waste [11–13]. Furthermore, with increasing emphasis on eco-friendly construction materials, these findings are significant. They potentially offer a route for integrating waste materials and developing high-performance, environmentally sound binding agents. The importance of this study lies in both its contribution to material science and its practical application in green buildings. Understanding how additives can enhance traditional materials, it aims to link sustainability with construction efficiency, providing useful insights for future research and industrial applications [14–17].

The recent investigations into gypsum-based composite materials have paid increasing attention to the application of various waste and by-products as additive elements to improve their physical and mechanical performance. One of such additives, which received significant attention during recent years, is reactive silica extracted from rice husk ash owing to high pozzolanic properties and its positive impact on matrix densification. As indicated by recent research findings, the silica content derived from rice husk ash also leads to pore refining and reinforcement via reaction with other compounds, whereas the use of both rice husk ash and microsilica together results in more efficient compactness and improved mechanical properties [18,19]. Additionally, sodium silicate has been extensively studied as an activator and an additional source of silica, promoting the formation of secondary reaction products and decreasing matrix porosity. The importance of regulating the alkalinity of the medium in which gypsum-based waste binder materials operate is shown by Gou et al. [20]. Finally, increased attention has been paid recently to the use of agricultural waste products as waste-derived admixtures in various construction materials. Among those, orange peel powder containing substantial amounts of pectin and natural sugars was proven to be potentially effective as a natural set-retarding admixture. Moreover, there has been much interest regarding the use of orange peel as a waste source due to its availability, rich content in pectin, and possible usage in the engineering field [21].

However, the synergistic effects of waste-based silica, alkali activators, and agricultural products in combination have not been studied sufficiently within the context of a single gypsum system. Though many studies have looked at the feasibility of modifying gypsum by using different kinds of wastes, individually, very little effort has gone into finding out what happens when more than one element is added together. This paper intends to improve mechanical characteristics of gypsum binders by applying reactive silica, sodium silicate solution, and ground orange peel powder (GOPP). The effects of each element and their combination in gypsum binder will be examined in regard to the setting process, compressive and flexural strengths, water resistance, and microstructure formation. As far as the available literature goes, very few studies have been conducted regarding the joint use of reactive silica derived from rice husk ash, sodium silicate derived from recycled glass waste, and GOPP, which serves as a natural set retarding agent, in a gypsum matrix. The uniqueness of this research study is based on assessing the effect of these three different additives on gypsum composites.

## 2. Materials and methods

These components, preparation techniques, and testing approaches help to investigate the additions to gypsum binders. Using industrial and agricultural residues, such as ground orange peel powder (GOPP), sodium silicate solution from recycled glass, and reactive silica from rice husk ash, this study improved the mechanical strength, water resistance, and setting behavior of gypsum-based composites. Many mixed forms have been investigated to assess the physical and chemical characteristics and dosages of these additives. The setting time, compressive strength, flexural strength, and water absorption were examined using standardized testing. Scanning electron microscopy (SEM) was used to investigate the morphological changes and bonding mechanisms of the modified matrix. The experimental foundation for the investigation was created by careful material selection and testing.

### 2.1 Materials

The main matrix material was mechanical gypsum plaster (G) purchased from a nearby Iraqi producer. Because it is inexpensive and simple to use, this type of plaster is frequently used in Iraq for wall and ceiling finishing. Partially calcined natural gypsum (calcium sulfate hemihydrate, CaSO_4_·0.5H_2_O) constitutes most of the plaster.

A chemically defined sodium silicate solution with the molecular formula Na_2_SiO_3_•9H_2_O was used as a multipurpose additive. With 28.7 weight percent SiO_2_, 8.9 weight percent Na_2_O, and 62.4 weight percent H_2_O, the solution had a SiO_2_/Na_2_O molar ratio of 3.22 and a hydration number of 9 moles of H_2_O per mole of Na_2_SiO_3_. This composition guarantees strong alkalinity (pH ~ 11.7), moderate viscosity (~240 cP at 25°C), and efficient reactivity with calcium-based binders. Sodium silicate was prepared in-house from recycled soda-lime glass waste, which normally contains 75 wt percent SiO_2_, 14 wt percent Na_2_O, and trace amounts of CaO and other oxides, to reduce the consumption of conventional raw materials and increase resource efficiency. After being finely ground (less than 45 μm), the waste glass was mixed with a 10 M sodium hydroxide solution and heated to 90 °C for two hours while being constantly stirred. To avoid carbonation, the resultant liquid sodium silicate was filtered and stored in airtight containers [22]. The addition of sodium silicate led to reduced water absorption and changes in the setting process because of the formation of other compounds that contain silicate in their composition and the resulting densification of the pores within the samples. This factor allowed the preparation of a composite material with a denser microstructure containing fewer pores, thus leading to better properties. Using a combination of acid leaching and regulated heat treatment, high-surface-area amorphous silica has been produced from agricultural waste, specifically from Iraqi rice husks. After thorough cleaning with deionized water to remove dust and soluble organic matter, the raw rice husks were oven-dried for 24 h at 105 °C. Subsequently, the dried husks were immersed in 0.1M hydrochloric acid (HCl) at 80 °C for two hours while being constantly stirred to remove metallic impurities such as Fe, K, Ca, and Mg. The husks were rinsed with deionized water until they reached neutral pH after leaching and then dried again.

**Fig 1:**
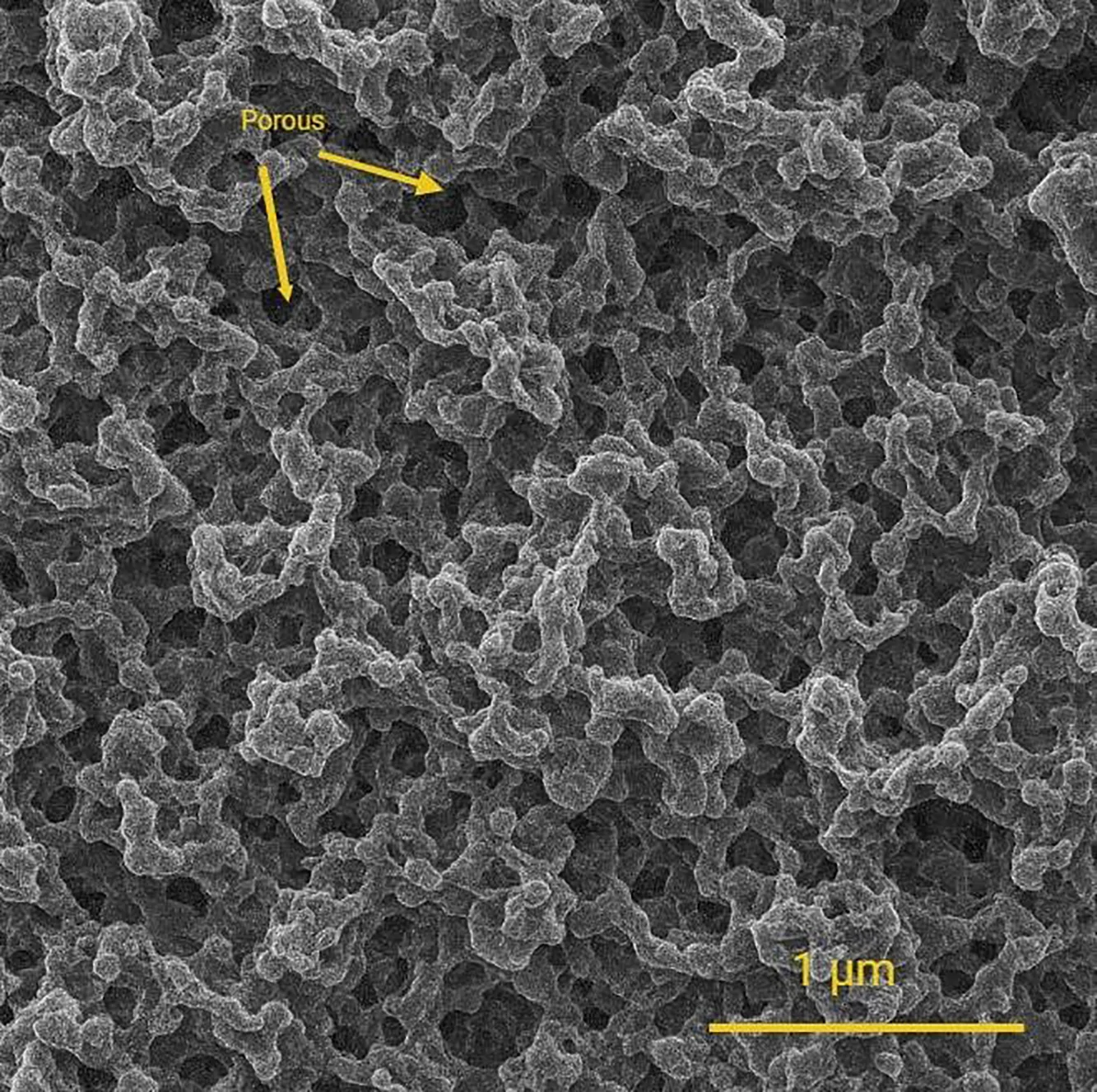
Microstructure of amorphous silica.

To eliminate the carbonaceous content while maintaining the amorphous structure of the resulting silica, the clean husks were calcined in air at 600 °C for two hours in a muffle furnace. After grinding and sieving to a size less than 75 µm, light-gray rice husk ash (RHA) was obtained. The produced silica exhibited exceptional qualities appropriate for cutting-edge uses: it had a Brunauer–Emmett–Teller (BET) surface area of 320 m^2^/g and contained approximately 94 wt percent SiO_2_. Scanning electron microscopy (SEM) revealed a highly porous morphology, as illustrated in Fig 1, and X-ray fluorescence (XRF) analysis validated the chemical composition. This eco-friendly process offers a low-cost method for producing high-value silica from agricultural waste, and is an appealing alternative for improving the functionality of cementitious composites and other functional materials [23].

For gypsum-based binders, ground orange peel powder (GOPP) is used as a natural and biodegradable set-retarding agent. After being gathered from local citrus processing waste, the orange peels were cleaned thoroughly, dried in an oven at 60 °C, and ground into a fine powder that could pass through a 75 μm sieve. Orange peels have a high amount of organic matter, with a total sugar and pectin content of approximately 33% of the dry weight, according to chemical characterization using XRF and the supporting literature. These include disaccharides, such as sucrose, and monosaccharides, such as fructose and glucose, all of which are known to affect the hydration kinetics of calcium-based binders.

These sugars and pectin adhere to the surface of hydrating particles to create transient barriers that prevent ion dissolution and delay the formation of hydration products, such as calcium silicate hydrates (C-S-H) or calcium sulfate dihydrate (CaSO_4_·2H_2_O), thereby improving workability and delaying stiffening. This effect is particularly beneficial in systems that are set quickly, such as highly reactive cementitious matrices or gypsum plasters. Utilizing citrus waste, such as orange peels, helps achieve sustainability objectives by reducing the dependency on artificial chemical retarders and valuing agro-industrial byproducts. The compatibility of the materials used in the construction was further confirmed by the fact that the presence of minor inorganic elements (K, Ca, and Mg), as identified by XRF, did not negatively affect the hydration process.

### 2.2 Mix design

Nine different mixtures, designated G1–G9 in Table 1, were created to examine the impact of the combined additives on the functionality of the gypsum-based composites. Three main factors were included in the mixed design parameters: the amount of reactive silica, amount of ground citrus peel powder (GCPP), and dosage of sodium silicate solution. Two levels of silica incorporation (5% and 10% by binder weight) and four levels of citrus peel powder addition (0%, 0.5%, 1%, and 2%) were used. Except for the additive-free control sample (G1), the sodium silicate solution was fixed at 20% of the binder weight in all modified mixtures (G2–G9). All mixtures were maintained at a water-to-binder (W/B) ratio of 0.4, except for G1, which required a slightly higher ratio of 0.5 to achieve workable consistency without the use of chemical additives. A decrease in the water-to-binder ratio in the modified mixes was required owing to the inclusion of sodium silicate solution, which increased the liquid component in the mixes and affected the workability of the gypsum paste. Thus, the chosen amount of water was selected keeping this in mind.

**Table 1:** The detailed composition of each mixture.

Mix ID	Silica (%)	Citrus Peel (%)	Sodium Silicate Solution (% wt of binder)	Water/Binder Ratio
G1	0	0	0	0.5
G2	5	0	20	0.4
G3	10	0	20	0.4
G4	5	0.5	20	0.4
G5	10	0.5	20	0.4
G6	5	1	20	0.4
G7	10	1	20	0.4
G8	5	2	20	0.4
G9	10	2	20	0.4

The individual and combined effects of reactive silica and organic retarders on the mechanical strength, water absorption, and setting behavior of gypsum composites can be systematically assessed using this matrix of mixes.

### 2.3 Experimental tests in this study

The physical, mechanical, and microstructural characteristics of the gypsum-based composites were assessed using a battery of standardized laboratory tests. A Vicat apparatus was used to measure the initial and final setting times of the gypsum-based materials in accordance with ASTM C472 [24]. This test elucidated the effects of citrus peel powder and sodium silicate on the workability and hydration behavior of the mixtures. A universal testing machine was used to measure the compressive strength of the 50 mm cube specimens in accordance with ASTM C472 [24]. After seven days of air curing, the specimens were tested to determine how the additives affected the ability of the hardened plaster to support the loads. A three-point bending setup based on the methods outlined in ASTM C348 [25], which was initially created for hydraulic cement mortars but provides an appropriate comparative methodology for brittle gypsum composites, was used to evaluate the flexural strength. Even though ASTM C348 [25] has been designed for the evaluation of hydraulic cement mortars, it can be deemed as appropriate for the current study because the goal here was to conduct an assessment of the flexural performance of binder-modified by reactive silica produced from rice husk ash that would have engineering applications in the future. In addition, the use of rice husk ash makes the composition and appearance of the binder highly non-homogenous, thus distinguishing it from ordinary white gypsum used in the manufacture of gypsum boards. Hence, it is possible to state that ASTM C348 is applicable when analyzing brittle composite materials. However, ASTM C472 [24] lacks a standard method for this purpose. The tensile response and flexural resistance of the modified mixtures were measured using prism samples with dimensions of 40 mm × 40 mm × 160 mm. ASTM C472 [24] was used to evaluate the water absorption, which entails submerging oven-dried specimens in water and calculating the mass increase as a percentage. This technique made it possible to evaluate the porosity and moisture infiltration, especially considering the sealing properties of reactive silica and sodium silicate. Scanning electron microscopy (SEM) was used to further analyze the microstructural features of the selected samples. SEM is a crucial tool for visualizing the internal structure and particle interactions within composites. However, it is not specifically covered by ASTM standards. The images provided crucial support for the interpretation of the macroscopic performance by revealing the morphology of the gypsum matrix, distribution of additives, and formation of hydration products. In addition, X-ray diffraction (XRD) analysis was carried out on some samples to determine the crystallographic phases of gypsum composites and the effect of reactive silica, sodium silicate, and orange peel powder on the formation of phases. The results of the XRD analysis were combined with the findings obtained from the SEM analysis to gain more insight into the structural changes taking place in the gypsum composites. To guarantee consistency and reduce random errors, three parallel specimens of each mixture were analyzed for statistical verification of all experimental data. The dependability and reproducibility of the results are demonstrated by the inclusion of the standard deviation values (±SD) in Table 2. A total of 108 samples were prepared and tested. Three samples were prepared for the compressive strength test, three for the flexural strength test, and three for the water absorption test for each of the nine mixes. Moreover, the setting times were measured in triplicate for each mix based on the ASTM C472 standards. It was found that the triplicate measurements made helped ensure more reliable results.

**Table 2:** Results of the tests in this study.

Mix ID	Setting time (min.) ±SD	Compressive Strength (MPa) ±SD	Flexural strength (MPa) ±SD	Water absorption % ± SD
G1	11 ± 0.6	7.2 ± 0.3	1.36 ± 0.05	21.50 ± 0.9
G2	8 ± 0.6	8.6 ± 0.4	1.81 ± 0.07	11.05 ± 0.5
G3	6 ± 0.6	9.3 ± 0.4	2.04 ± 0.08	6.72 ± 0.3
G4	17 ± 1.0	10.9 ± 0.5	1.90 ± 0.07	8.80 ± 0.4
G5	12 ± 0.6	11.6 ± 0.5	2.18 ± 0.09	5.97 ± 0.3
G6	33 ± 1.0	14.1 ± 0.6	2.19 ± 0.09	6.89 ± 0.3
G7	27 ± 0.6	16.9 ± 0.7	2.35 ± 0.10	5.41 ± 0.2
G8	59 ± 1.0	15.2 ± 0.6	2.08 ± 0.08	7.21 ± 0.3
G9	48 ± 1.5	15.5 ± 0.7	2.21 ± 0.09	5.50 ± 0.2

## 3. Results and discussion

Table 2 presents the results of the compressive strength tests conducted on the gypsum-based composites. Because unmodified gypsum is naturally brittle and porous, reference mix G1, which had no additives, had the lowest compressive strength at 7.2 MPa. Mixes G2 and G3 saw a moderate increase in strength to 8.6 MPa and 9.3 MPa, respectively, upon the addition of sodium silicate and reactive silica. The high surface reactivity and microfilling effect of reactive silica, which aids in pore refinement and the formation of new calcium silicate hydrate (C–S–H) phases, are responsible for this improvement [26]. The compressive strength of the silica-based system further increased with the addition of citrus peel powder. Mixtures G4 and G5, containing 0.5% citrus peel powder and 5% and 10% reactive silica, respectively, achieved compressive strengths of 10.9 MPa and 11.6 MPa. The natural sugars in citrus peels slow the setting time, providing a C-S-H gel with more time to form and produce a denser and more cohesive matrix [27]. Mixtures G6, G7, G8, and G9, which included 1%–2% powdered citrus peel, had the highest compressive strengths. The highest value, 16.9 MPa, was attained by G7, followed by G9 (15.5 MPa), G8 (15.2 MPa), and G6 (14.1 MPa). These findings demonstrate a strong synergistic relationship between the prolonged development of C-S-H phases and delayed hydration caused by citrus peel sugars and pectin. More complete reactions between calcium ions, reactive silica, and sodium silicate were made possible by the extended setting time [28]. Overall, the results indicated that the addition of reactive silica, sodium silicate solution, and powdered citrus peel significantly increased the compressive strength of the gypsum composites. Instead of being a disadvantage, delayed hydration is a helpful mechanism that enhances microstructural densification and crystal growth. The chemical environment produced by the addition of sodium silicate was quantitatively assessed to support these findings. The mix design included a 20% sodium silicate solution and 40% water by weight of gypsum. The effect was confirmed by a simple calculation: Six grams of pure Na_2_SiO_3_ was obtained from 20 g of sodium silicate solution that contained 30% active Na_2_SiO_3_. The moles of Na_2_SiO_3_ were 0.049 (6 ÷ 122), which yielded a molarity of approximately 0.91 mol/L when considering a total liquid volume of 54 mL (40 g added water + ~14 g from the solution). This concentration caused Na_2_SiO_3_ to hydrolyze and release OH^−^ ions, which raised the pH to approximately 11.5–12.0, which was sufficient to initiate the pozzolanic reaction. This enables reactive silica to join with the released calcium ions of gypsum to create secondary C-S-H gels, which thicken the matrix and improve interparticle adhesion. This process explains the steady increase in strength, particularly in G5–G9, where the combination of reactive silica, sodium silicate, and powdered citrus peel produced ideal gel formation and improved the matrix cohesion of the composite. These results align with those of Hamadi et al. (2022) [29], who found that similar molarity sodium silicate solutions efficiently raised the pH to levels that encouraged C-S-H formation in cementitious systems, thereby improving densification and mechanical performance.

### 3.1 Results of flexural strength

Table 2 presents the flexural strength results for the modified gypsum mixtures. In accordance with the brittle nature of unreinforced gypsum, the reference mix G1 exhibited the lowest flexural strength at 1.36 MPa. The flexural strength values increased to 1.81 MPa and 2.04 MPa, respectively, owing to the addition of reactive silica and sodium silicate to G2 and G3. These enhancements were ascribed to the improved bonding and densification effects of reactive silica, which promoted a more refined microstructure and increased the resistance of the gypsum matrix to bending forces [26]. Mixes G4 and G5 exhibited mixed behavior when 0.5% citrus peel powder was added; G4 slightly decreased to 1.90 MPa, while G5 increased to 2.18 MPa. This variation might result from the dual function of sugars derived from citrus, which initially slows the hydration reaction but then improves the formation of calcium-silicate-hydrate (C-S-H) phases that improve tensile bridging [27]. The flexural strength increased steadily with additional citrus peel content up to 1%–2% (G6–G9), peaking at 2.35 MPa in mix G7. This implies that the enhanced fiber-like interaction of the organic content within the matrix and the delayed hydration kinetics work in concert. Furthermore, the development of secondary silicate-based gels in the presence of sodium silicate enhances the resistance to bending loads and decreases the propagation of microcracks [28]. Overall, the flexural performance of the gypsum composites was significantly enhanced by the addition of reactive silica, sodium silicate solution, and powdered citrus peel. According to the data, a useful method for improving the tensile-related mechanical properties is to alter the hydration dynamics and microstructure using multifunctional additives. Flexural strength enhancement occurs because of multiple factors that work synergistically. Reactive silica serves as a microfiller and nucleating agent by providing nucleation centers for hydration products, resulting in a densified and uniform structure. Sodium silicate serves as an additive that promotes the generation of secondary C-S-H phases and improves particle binding and crack resistance. Similarly, Elrefaei and Batova [28] showed that silica-containing additives facilitate the densification of the matrix and improve its mechanical characteristics. Moreover, organic compounds, such as natural sugars and pectins, included in orange peel powder slow down the process of hydration and help develop hydration products slowly and evenly without causing any defects inside the matrix. It can be assumed that such retardation of hydration results in better stress transmission and thus leads to enhanced flexural strength values. This phenomenon has also been observed in previous studies conducted on biological building materials [27,30].

### 3.2 Results of water absorption

The water absorption values of the gypsum-based mixtures are listed in Table 2. Because the untreated gypsum was naturally porous, the control mix (G1) exhibited the highest absorption rate of 21.5%. When reactive silica and sodium silicate were added to G2 and G3, the absorption decreased significantly to 11.05% and 6.72%, respectively. The fine particle size and pozzolanic activity of reactive silica, which densifies the matrix by reacting with calcium ions to form C–S–H gels, contribute to this reduction. By blocking the capillary pores, these gels successfully increase water resistance [26,31]. Absorption rates of 7.80% and 5.97% were demonstrated by mixes G4 and G5, respectively, which contained 0.5% ground orange peel powder (GOPP). The natural sugars and pectin in the orange peels slowed the hydration process and encouraged the slow development of C-S-H structures. Additionally, these substances function as organic fillers that improve moisture resistance by decreasing pore connectivity [27,30]. The absorption performance was further stabilized by the higher GOPP concentrations in G6–G9, with values ranging from 5.41% to 6.71%. G7 exhibited the lowest water absorption capacity (5.41%). This pattern demonstrates how reactive silica, sodium silicate, and bioactive compounds derived from citrus peels work together to improve the pore structure, lower permeability, and promote the formation of less hygroscopic C–S–H phases. The efficacy of these combinations is consistent with previous research showing that the moisture durability of gypsum systems can be considerably increased using natural and nanoscale additives [28,32]. These results indicate that it is feasible to create long-lasting and ecologically friendly gypsum-based composites by utilizing industrial and agricultural wastes, such as reactive silica from rice husk ash and citrus peels.

### 3.3 Microstructural and phase analysis (SEM and XRD)

**Fig 2:**
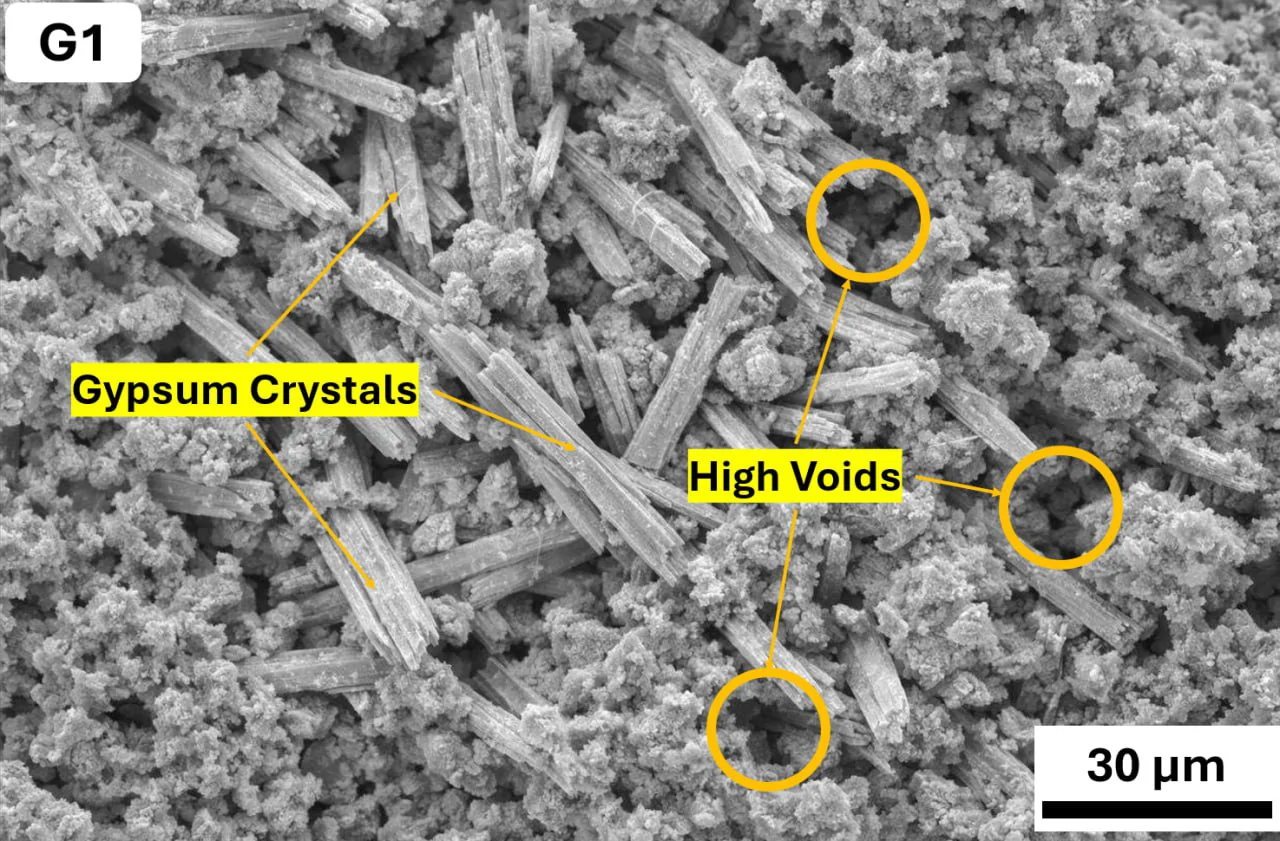
SEM of the G1 mixture.

**Fig 3:**
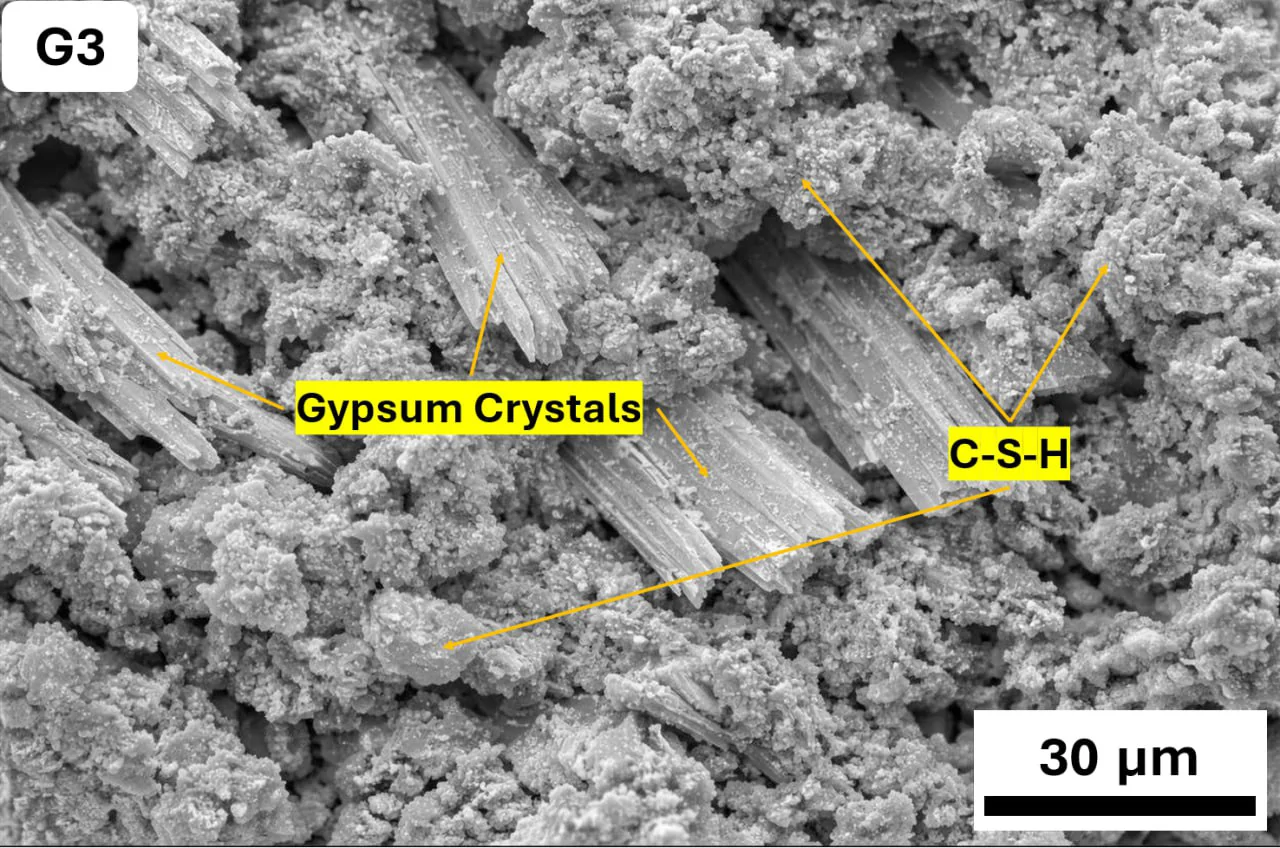
SEM of the G3 mixture.

**Fig 4:**
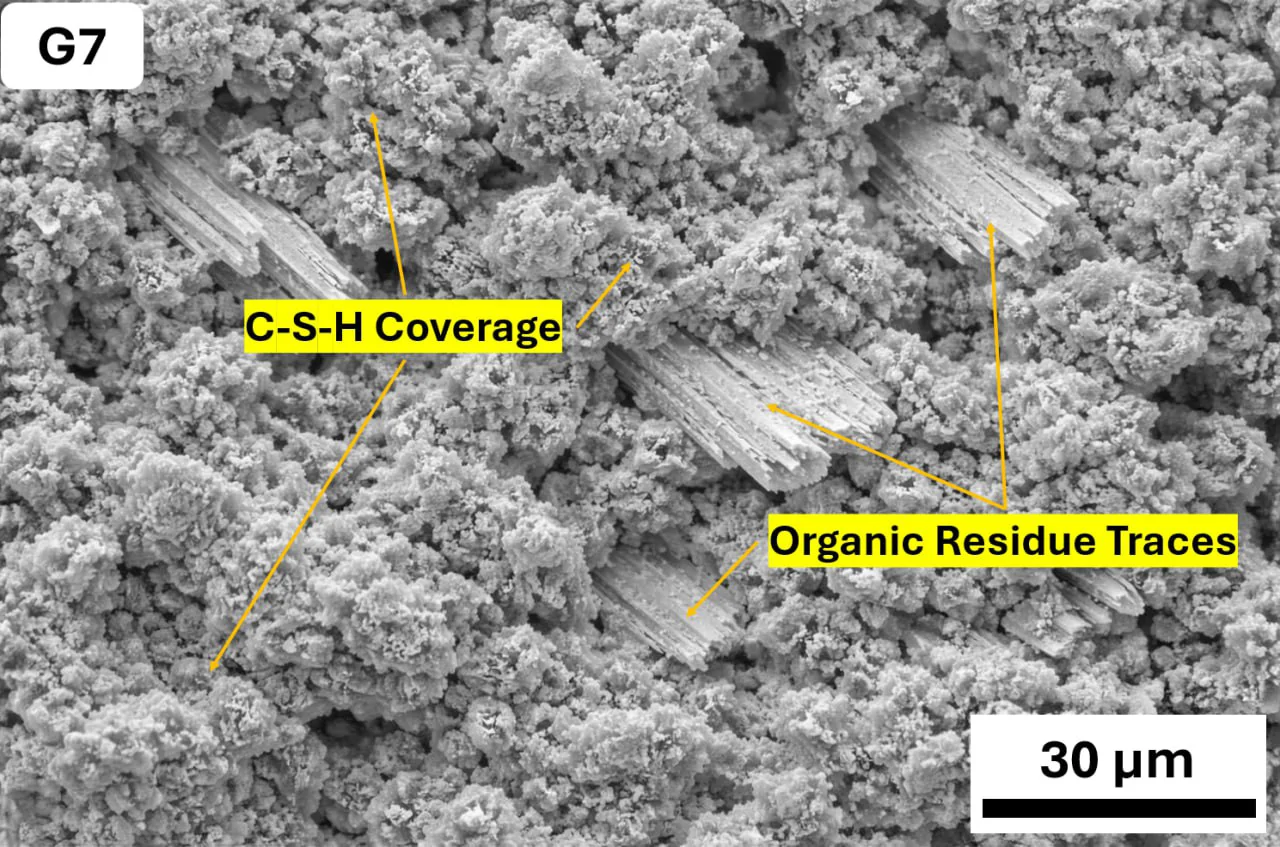
SEM image of the G7 mixture.

**Fig 5:**
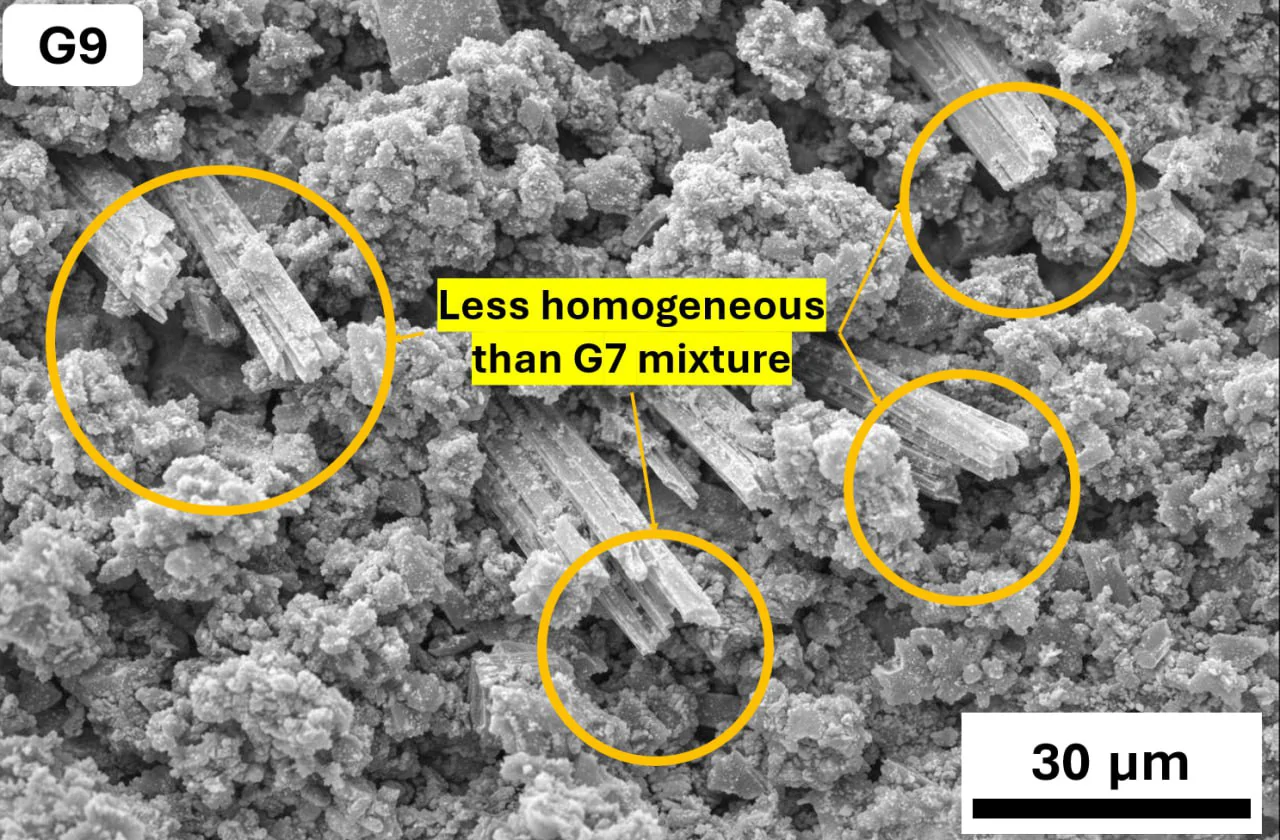
SEM image of the G9 mixture.

**Fig 6:**
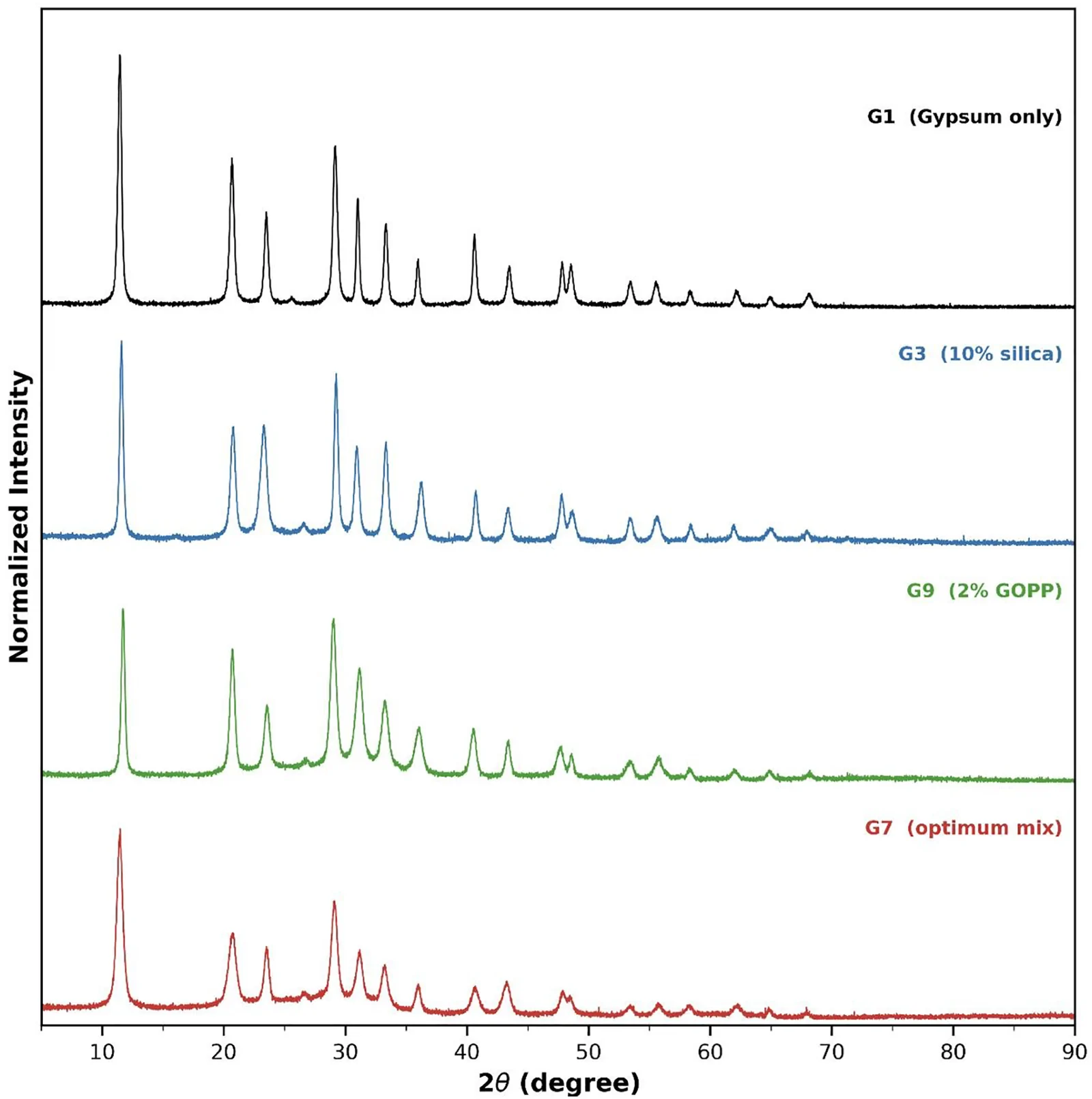
XRD analysis results for the selected gypsum-based composites (G1, G3, G7, and G9) demonstrating phase formation and the effect of reactive silica, sodium silicate, and orange peel on the crystalline structure development.

SEM micrographs of the gypsum-based composites are shown in Figs 2–5 (corresponding to mix G1 (Fig 2), G3 (Fig 3), G7 (Fig 4), and G9 (Fig 5), respectively) clearly showed differences in morphology, particle interconnectivity, and porosity. Further confirmation of the SEM analysis was obtained from the XRD findings, as depicted in Fig 6, showing the impact of reactive silica, sodium silicate, and ground orange peel powder on the gypsum system. These variations demonstrate the effects of different additive combinations and dosages on the internal microstructure.

Long needle-like gypsum crystals (CaSO_4_·2H_2_O) were randomly oriented and distributed throughout the matrix in the microstructure of G1. In the absence of additives, crystals of gypsum hydrate very quickly and have larger, more irregular shapes. This leads to greater porosity and less binding between particles, which results in lower strength. The texture was extremely porous, with noticeable intercrystallite voids and little interlocking. This morphology is typical of traditional gypsum systems, where high water absorption and weak interparticle bonding result from rapid hydration and crystallization. The low compressive and flexural strengths of G1 and its high permeability can be explained by the rapid setting and limited densification caused by the absence of additives. The XRD pattern of G1 was dominated by gypsum peaks, suggesting that there were no substantial secondary reaction products.

The gypsum crystals were partially embedded in a continuous matrix of secondary gel phases in the densely packed structure observed in G3. SEM analysis indicated the formation of further reaction products due to the reactions of reactive silica and sodium silicate with the gypsum matrix. Gel formation is further encouraged by the addition of a sodium silicate solution (20 wt percent of binder), which serves as an activator and a source of silica. A finer pore structure and fewer voids were obtained. This is caused by the creation of reaction products that connect gypsum particles and fill capillary spaces, while sodium silicate and reactive silica work in concert to improve matrix integrity. The formation of a denser matrix with fewer interparticle voids and stronger bonding between hydration products contributed to the observed improvement in the mechanical strength. These findings were supported by the XRD tests, where it was seen that there was variation in the intensity of the peaks and additional phases formed from the altered mixtures.

Among all the mixes, the microstructure was more densely packed in G7, which had more evenly distributed products of reaction and low pore density. Amorphous or semi-amorphous reaction products heavily coat the crystal surfaces. Natural sugars, such as fructose, which adsorb on crystal surfaces and postpone nucleation, were introduced using citrus peel powder (1 wt percent). This delay enabled better silica gel distribution and prolonged reaction development. The amounts of sodium silicate and reactive silica were the same as those in G3; however, better packing and less premature stiffening were made possible by the delayed kinetics caused by organic interactions. Sufficient retardation was achieved by the optimal use of citrus peel at 1% without impeding the formation of advantageous hydration products. XRD of the G7 sample further substantiated the SEM results in that it had the most developed phase structure compared to the other mixtures tested. G7 is the best-performing mix in terms of strength and moisture resistance because the resulting matrix is more cohesive, has fewer defects, and exhibits superior mechanical performance.

Elongated gypsum crystals scattered throughout an uneven gel-like matrix are visible in Mix G9, which is 10% reactive silica + 2% citrus peel powder + 20% sodium silicate. The higher dosage of citrus peel powder (2 wt percent) causes over-retardation, delaying the binding phase past the ideal bounds, even though reaction products are still visible. An overabundance of organic materials can lead to uneven gel dispersion and localized inhibition of crystallization. The gel matrix was not as homogeneous as that in G7, and some gypsum crystals were unduly exposed. The XRD results supported these findings, as they indicated that too much retardation hindered the positive phase formation observed for G7. Increasing the amount of citrus peel improves workability and postpones setting, but also prevents the reaction network from developing optimally. Because of the dispersed and partially interrupted reaction front, this mix performed better than G1; however, it was less homogeneous and produced a lower mechanical performance than G7.

### 3.4 Comparing this study to others

The results of this investigation showed that the mechanical and physical performances of gypsum binders were significantly enhanced by the addition of sodium silicate, reactive silica, and citrus peel powder (GOPP). The optimized mixture (G7) outperformed the control mixture by approximately 135% and 73%, respectively, achieving a compressive strength of 16.9 MPa and a flexural strength of 2.35 MPa. Furthermore, the water absorption decreased considerably from 21.5% to 5.4%, demonstrating the improved density and moisture resistance of the composite matrix. These enhancements outperformed the findings of several earlier investigations that used agricultural waste materials in gypsum matrices. For example, Fernandez et al. (2024) [27] found that adding ground pistachio shells increased the compressive strength by approximately 160%; however, there was only a slight decrease in capillary absorption. However, despite offering modest improvements in thermal insulation, Ataie (2018) [33] and Tichi and Khatiri (2024) [34] found that adding rice-straw fibers reduced the compressive strength. Singh et al. (2023) [32] found that foamed gypsum blocks containing rice straw exhibited a 53% increase in compressive strength, a 25% improvement in flexural strength, and a roughly 18% decrease in water absorption. According to Shaikh et al. (2025) [35], the addition of lignin and nanocellulose usually increases the compressive and flexural strengths by only 15–20% when compared to cement-based systems. Therefore, the strength, ductility, and water resistance of the GOPP–reactive silica–sodium silicate composite suggested in this work show a more balanced and synergistic improvement. The high environmental relevance of citrus waste is further highlighted by the valuation of this formulation, which promotes the shift to more sustainable and green building materials.

### 3.5 Practical implications and limitations

In summary, the findings of the current study indicate the possibility of applying reactive silica, sodium silicate, and ground orange peel powder to improve the performance of gypsum composites. Specifically, the mixtures prepared during this experiment exhibited enhanced mechanical properties, low water absorption, and regulated setting characteristics compared to the reference mixture. Therefore, it may be assumed that the materials suggested in this study can be used as components of interior finishing materials, decorative gypsum products, and lightweight building constructions. Additionally, because rice husk ash, sodium silicate made of recycled glass, and orange peel powder were used in the preparation of the mixtures, the application of these materials may lead to the efficient valorization of industrial waste streams, thus contributing to more sustainable construction practices. Nonetheless, a dedicated methodology is required to estimate the sustainability of materials.

However, there are several disadvantages that must be emphasized as well. First, in the course of this research, much attention was paid to the short-term mechanical properties, water absorption, and setting behavior. The lack of data on durability characteristics implies that the long-term behavior of new materials has not yet been evaluated. In addition, even though some physicochemical analysis methods, such as SEM and XRD, have been applied, it may also make sense to conduct FTIR, TGA, and porosity analysis. Moreover, since the liquid composition in the developed mixtures differed from the liquid used in the control mixture owing to sodium silicate, one should take it into account when interpreting the results. Finally, additional studies are required to evaluate the durability of new mixtures.

## 4. Conclusions

This study illustrates how the addition of reactive silica, sodium silicate solution, and ground orange peel powder (GOPP) to gypsum-based composites improves their mechanical properties and moisture resistance. Owing to its porous structure and the absence of additional materials, the control mix (G1) exhibited the highest water absorption and lowest performance in terms of compressive and flexural strengths. The addition of reactive silica and sodium silicate solutions significantly increased the compressive and flexural strengths while decreasing the water absorption owing to the refinement of the pore structure and the formation of additional silicate-containing reaction products, as supported by SEM and XRD analyses. Additionally, GOPP acted as a waste-derived set-retarding additive by delaying the setting process and promoting a more favorable development of the gypsum matrix. Mixtures containing 10% reactive silica, 20% sodium silicate solution, and 1% GOPP (G7) exhibited the best overall performance, demonstrating an effective balance between mechanical strength and moisture resistance. The findings from the SEM and XRD analyses indicate that the use of reactive silica and sodium silicate had a positive impact on the microstructure owing to better density and homogeneity, while GOPP improved the dispersion of the reaction products within the matrix. This was evidenced by the enhanced mechanical and physical properties of the composite material. It can be seen that the use of silica from rice husk ash, sodium silicate obtained from recycled glass, and orange peel powder has a lot of value when it comes to making gypsum composites. This approach is helpful in making proper use of industrial and agricultural waste products.
